# Enhanced Cellular Uptake in an Electrostatically Interacting Fucoidan–L-Arginine Fiber Complex

**DOI:** 10.3390/polym13111795

**Published:** 2021-05-29

**Authors:** Vinothini Arunagiri, Hsieh-Chih Tsai, Haile Fentahun Darge, Endiries Yibru Hanurry, Chang Yi Lee, Juin-Yih Lai, Szu-Yuan Wu

**Affiliations:** 1 Graduate Institute of Applied Science and Technology, National Taiwan University of Science and Technology, Taipei 106, Taiwan; applesssswcc@gmail.com (V.A.); fentahunhailebdu@gmail.com (H.F.D.); Endris_Yibru@dmu.edu.et (E.Y.H.); m10722602@gapps.ntust.edu.tw (C.Y.L.); jylai@mail.ntust.edu.tw (J.-Y.L.); 2 Advance Membrane Materials Center, National Taiwan University of Science and Technology, Taipei 106, Taiwan; 3 R&D Center for Membrane Technology, Chung Yuan Christian University, Chungli, Taoyuan 320, Taiwan; 4 College of Medicine and Health Science, Bahir Dar University, Bahir Dar 79, Ethiopia; 5 Department of Food Nutrition and Health Biotechnology, College of Medical and Health Science, Asia University, Taichung 413, Taiwan; 6 Big Data Center, Lo-Hsu Medical Foundation, Lotung Poh-Ai Hospital, Yilan 256, Taiwan; 7 Division of Radiation Oncology, Department of Medicine, Lo-Hsu Medical Foundation, Lotung Poh-Ai Hospital, Yilan 256, Taiwan; 8 Department of Healthcare Administration, College of Medical and Health Science, Asia University, Taichung 413, Taiwan; 9 Cancer Center, Lo-Hsu Medical Foundation, Lotung Poh-Ai Hospital, Yilan 256, Taiwan; 10 Graduate Institute of Business Administration, Fu Jen Catholic University, Taipei 242, Taiwan; 11 Centers for Regional Anesthesia and Pain Medicine, Taipei Municipal Wan Fang Hospital, Taipei Medical University, Taipei 110, Taiwan

**Keywords:** L-arginine, fucoidan, cyanine 3 dye

## Abstract

Fucoidan is an abundant marine sulfated polysaccharide extracted from the cell wall of brown macroalgae (seaweed). Recently, fucoidan has been highly involved in various industrial applications, such as pharmaceuticals, biomedicals, cosmetics, and food. However, the presence of a sulfate group (negative surface charge) in the fucoidan structure limits its potential and biological activity for use in biomedical applications during cellular uptake. Thus, we aimed to improve the uptake of fucoidan by using an L-arginine uptake enhancer within an in vitro study. A Fucoidan–L-Arginine (Fuc-L-Arg) fiber complex was prepared via α-helical electrostatic interactions using a freeze-drying technique and confirmed using field-emission scanning electron microscopy, Fourier transform infrared spectroscopy, and nuclear magnetic resonance spectroscopy. In addition, fucoidan was conjugated with cyanine 3 (Cy3) dye to track its cellular uptake. Furthermore, the results of Fuc-L-Arg (1:1, 1:2.5) complexes revealed biocompatibility >80% at various concentrations (5, 10, 25, 50, 100 µg/mL). Owing to the higher internalization of the Fuc-L-Arg (1:5) complex, it exhibited <80% biocompatibility at higher concentrations (25, 50, 100 µg/mL) of the complex. In addition, improved cellular internalization of Fuc-L-Arg complexes (1:5) in HeLa cells have been proved via flow cytometry quantitative analysis. Hence, we highlight that the Fuc-L-Arg (1:5) fiber complex can act as an excellent biocomplex to exhibit potential bioactivities, such as targeting cancers, as fucoidan shows higher permeability in HeLa cells.

## 1. Introduction

Fucoidan is an abundant marine sulfated algal hetero-polysaccharide, and a biocompatible material mainly consisting of L-fucose and sulfate groups [1]. The structure of fucoidan comprises an α-1,3-backbone, or a repeating unit comprising disaccharides containing α-(1–3)- and α-(1–4)-bonded L-fucopyranose residues with additional branches attached at C2 positions. Like its high molecular weight form, low molecular weight (LMW) fucoidan (oligo-fucoidan) also exhibits various bioactivities such as anti-cancer, anti-viral, anti-oxidant, and anti-inflammatory activities. The above properties have attracted researchers to use fucoidan in order to study various applications, such as nanotechnology, drug delivery systems, tissue engineering, cancer therapy, wound dressing, biosensors, and water treatment. In addition, fucoidan has also received significant attention in the food and pharmaceutical industries owing to its advantageous therapeutic effects [2]. Several studies have shown that fucoidan combats cancer cell formation, development, and growth [1,3,4].

Sulfate is one of the main structural and chemical factors associated with biologically or physiologically active polysaccharides [5]. For example, heparin is clinically used as an anti-coagulant and its sulfate content is associated with its anti-coagulant activity [6]. In another case, the sulfonated residues of laminarians were found to be effective in preventing and treating ischemic cerebrovascular disease. Dextran sulfate is effective in inhibiting human immunodeficiency virus (HIV), and demonstrated the increased ability of over-sulfated fucoidan to bind to and inhibit basic fibroblast growth factor (bFGF). The number of sulfate groups in fucoidan contributes to the efficacy of its anti-angiogenic and anti-tumor activities. Furthermore, low molecular weight (LMW) fucoidan from *Ascophyllum nodosum* is involved in anti-coagulant and anti-proliferative activities, and these properties also depend on the number of sulfate groups [5,7,8]. The surface charge of a biomaterial is a significant factor regarding cellular uptake when considering potential therapeutic approaches. However, the extent of oral absorption of fucoidan is sometimes insufficient to assess its bioactivity, owing to its highly negative surface charge which results in poor membrane permeability [9,10].

The cell membrane has a negative surface charge due to the presence of glycosaminoglycan polysaccharide (GAG), heparin sulfate (HS), chondroitin sulfate (CS), and dermatan sulfate (DS) [11]. However, both the cell membrane and fucoidan have affinity towards positively charged compounds including cell-penetrating peptides (CPPs) [12,13,14,15]. The presence of the above groups repels the entry of negative surface charged fucoidan into the cell since it also contains a sulfate group. Thus, in our study, we chose L-arginine as an enhancer because it can act as a CPP [16,17,18] for greater cellular uptake of fucoidan. L-arginine is a semi-essential amino acid found in nuts and meats, and it acts as a substrate for the production of nitric oxide [1,5,8,11,12,13,14,15,16,19,20,21,22,23,24,25,26,27,28,29,30,31,32,33].

In the past five years, the novel concept of immuno-chemotherapy has been introduced using fucoidan to enhance the immune system’s action against cancer. However, there are no quality trials involving fucoidan in the treatment of human cancer because of its large molecular structure and the unavailability of an optimal route of administration owing to its poor bioavailability. Previous studies have reported that chitosan oligomers [15] and L-arginine [15,34] are novel absorption enhancers capable of increasing the intestinal absorption of fucoidan and gastro-intestinal absorption of heparin, respectively. Hence, in this study, we proposed a method for adding the absorption enhancer L-arginine to fucoidan in order to improve its cellular uptake.

## 2. Materials and Methods

### 2.1. Materials

LMW fucoidan (oligo-fucoidan) was extracted from *Laminaria japonica* seaweed and further prepared by Hi-Q Marine Biotech International Ltd. (molecular weight around 500–1500 Da). The oligo-fucoidan comprises of L-fucose (210.9 ± 3.3 μmol/g) and sulfate ester (38.9 ± 0.4% *w*/*w*) (New Taipei City, Taiwan). Cyanine 3 (Cy3) NHS ester was obtained from Lumiprobe (Hallandale Beach, FL, USA). L-arginine, L-ethyl-3-(3-dimethyl aminopropyl) carbodiimide (EDC), N-hydroxysuccinimide (NHS), ethylenediamine (EDA), pyridine, and dichloromethane (DCM) were obtained from Sigma-Aldrich (St. Louis, MO, USA). The cellulose dialysis membrane (molecular weight cutoff [MWCO]: 1000 Da) and deionized (DI) water used in the experiments were obtained from Orange Scientific (Chennai, India) and the Millipore water purification system was from MilliporeSigma (Burlington, MA, USA). HeLa cells (Thermo Fisher Scientific, Waltham, MA, USA), Dulbecco’s modified Eagle medium (DMEM), and fetal bovine serum (FBS) were obtained from the American Type Culture Collection (Rockville, MD, USA).

### 2.2. Preparation of the Fucoidan-L-Arginine (Fuc-L-Arg) Fiber Complex

Fucoidan and L-arginine were mixed together with different weight ratios of 1:1, 1:2.5, and 1:5, respectively. Further, the mixtures were stirred at 300 rpm for approximately 12 h, purified using a 1000 MWCO dialysis membrane for one day in a DI water environment, lyophilized, and characterized.

### 2.3. Conjugation of Oligo-Fucoidan to Cyanine 3 NHS Ester Dye (Fuc-Cy3) as a Biomarker to Track Cellular Uptake

First, 1 mmol of the oligo-fucoidan acid group was activated using 3 mmol EDC and 4 mmol NHS in 10 mL DCM solvent, followed by stirring for 12 h. To the activated acid group, 8 mmol of EDA and 200 µL pyridine were added and stirred for 24 h to aminate the fucoidan. Later, the aminated fucoidan was purified using a 1000 MWCO dialysis bag in a DI water environment for two days and lyophilized. Then, to the 18 mmol of aminated fucoidan, 0.2 mmol Cys3 NHS ester dye in 10 mL DCM was added and stirred for 24 h. Furthermore, the Cy3 NHS ester dye-conjugated fucoidan was purified using a 1000 MWCO dialysis bag in a DI water environment for two days, lyophilized, and characterized.

### 2.4. Characterization of the Fucoidan-L-Arginine Fiber Complex and Fucoidan-Cyanine 3 NHS Ester Dye

The Fuc-L-Arg complex and Fuc-Cy3 dye were characterized using attenuated total reflectance Fourier transform infrared (ATR FT-IR) spectroscopy (FT/IR-6700; JASCO Inc., Oklahoma City, OK, USA). Proton nuclear magnetic resonance (1H NMR) spectroscopy (AVANCE 500.163 MHz; Bruker, Billerica, MA, USA) using deuterium oxide as the solvent was also performed. Field-emission scanning electron microscopy (FE-SEM; JSM 6500F; JEOL, Tokyo, Japan) operating at 15.0 kV was conducted to observe the morphology of the Fuc-L-Arg complex. The conjugation of Fuc-Cy3 dye and Fuc-L-Arg was confirmed using ultraviolet–visible (UV–Vis) spectra measured using a JASCO V-650 spectrophotometer. The fluorescence intensity of the Fuc-Cy3 dye was assessed using a JASCO FP-8300 spectrophotometer equipped with a xenon lamp power supply and 1 cm path quartz cell at Excitation and Emission bandwidths of 10 nm, a response of 0.1 s, medium sensitivity, a data interval of 1 nm, and a scan speed of 1000 nm/min.

### 2.5. Cell Viability of the Fucoidan-L-Arginine Fiber Complex

The cell viability test was carried out for fucoidan, L-arginine, and the Fuc-L-Arg complex at different concentrations using an MTT assay in HeLa cells. Briefly, HeLa cells were seeded in 96-well plates at a density of 1 × 10^4^ cells/well in 100 µL DMEM and incubated for 24 h at 37 °C in a 5% CO_2_ environment. Then, the incubated cells were treated with 100 µL DMEM of different concentrations (5, 10, 25, 50 and 100 µg/mL) of pristine fucoidan, pristine L-arginine, and Fuc-L-Arg followed by incubation for 24 h at 37 °C in a 5% CO_2_ environment. After incubation, the medium was discarded and washed twice with 1× PBS. Next, 100 µL MTT solution was added to each well for 4 h of further incubation. Subsequently, the MTT solution culture medium was discarded carefully and followed by the addition of 100 µL dimethyl sulfoxide to dissolve the formazan crystals. Then, the plates were kept outside of the incubator for 20 min before measuring the absorbance at 570 nm. The percentage of cell viability of the material was calculated as follows:

$$Cell\;Viability=\frac{absorbance\;of\;the\;sample}{absorbance\;of\;control}\;\times \;100$$

### 2.6. In Vitro Qualitative Cellular Uptake Analysis of the Fuc-L-Arg Fiber Complex

Qualitative cellular uptake was determined using confocal fluorescence microscopy. At first, HeLa cells were seeded at a density of 1 × 10^5^ cells/well in a cover glass bottom dish. After 24 h, the culture media were replaced with fresh media containing control group Fuc-Cy3 (10 µg/mL) and different weight ratios of the Fuc-Cy3-L-Arg complex (1:1, 1:2.5, and 1:5) with 10 µg/mL concentration. Then, the cells were incubated for 4 h. After incubation, the medium was removed and washed with PBS three times, and then stained with 4′,6-diamidino-2-phenylindole (DAPI) for 20 min at room temperature, and fixed with 4% formalin solution. Images were viewed using fluorescence microscopy (iRiS^TM^ Digital Cell Imaging System; Logos Biosystems, Anyang, South Korea).

### 2.7. Quantitative Analysis of the Fuc-L-Arg Fiber Complex (Flow Cytometry)

To quantify the permeability of the Fuc-Cy3 dye and Fucoidan-Cyanine 3 NHS Ester dye-L-Arginine (Fuc-Cy3-L-Arg) (1:1, 1:2.5 and 1:5) complex, HeLa cells were seeded in 6-well plates at a density of 5 × 10^5^ cells/well. The cells were incubated at 37 °C in 5% CO_2_ environment for 24 h. Then, the incubated cells were treated with 10 µg Fuc-Cy3 dye and Fuc-Cy3-L-Arg (1:1, 1:2, and 1:5) complex followed by 4 h of incubation. Later, the cells were trypsinized and resuspended in 500 μL of PBS. For each sample, 5000 events were collected, and fluorescence was detected using fluorescence-activated cell sorting.

## 3. Results and Discussion

### 3.1. Confirmation of Fuc-L-Arg Fiber Complex Interactions Using Spectroscopy

**Figure 1:**
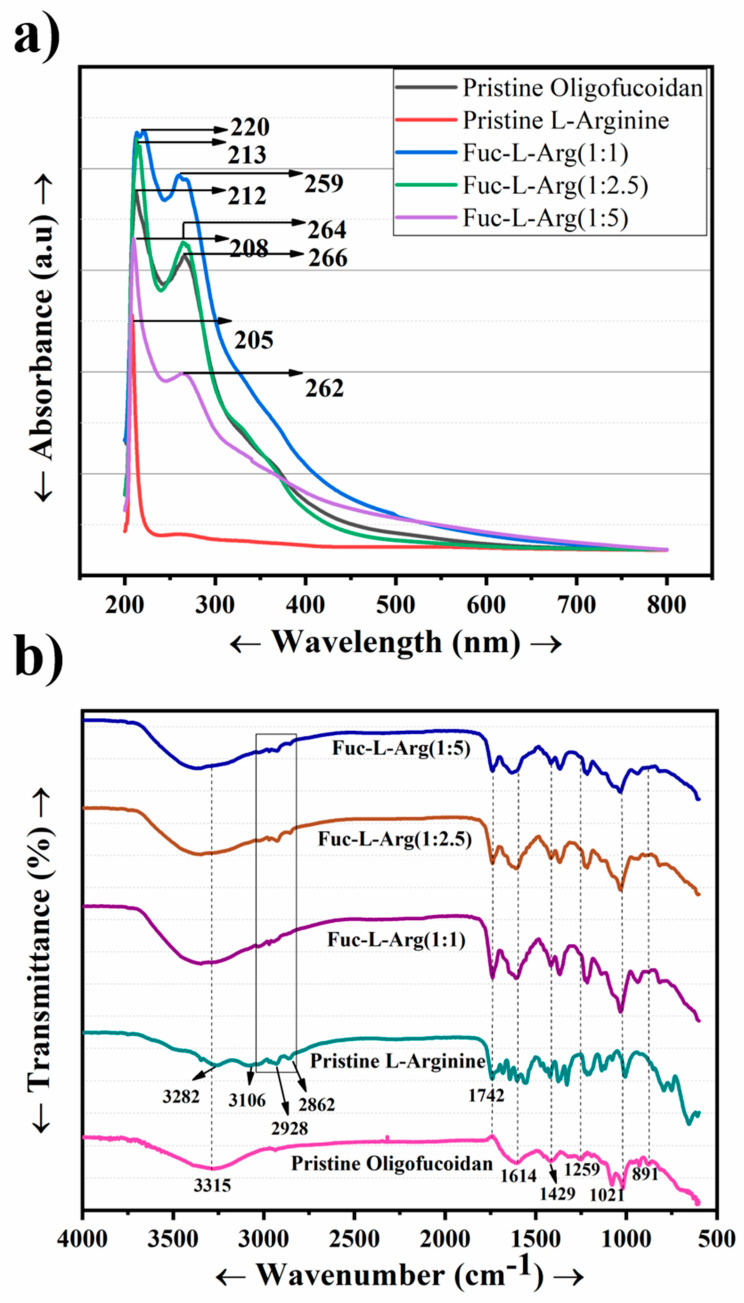
(**a**) UV absorbance of fucoidan, L-arginine, and their complexes; (**b**) FT-IR characterization of fucoidan, L-arginine, and their complexes.

**Figure 2:**
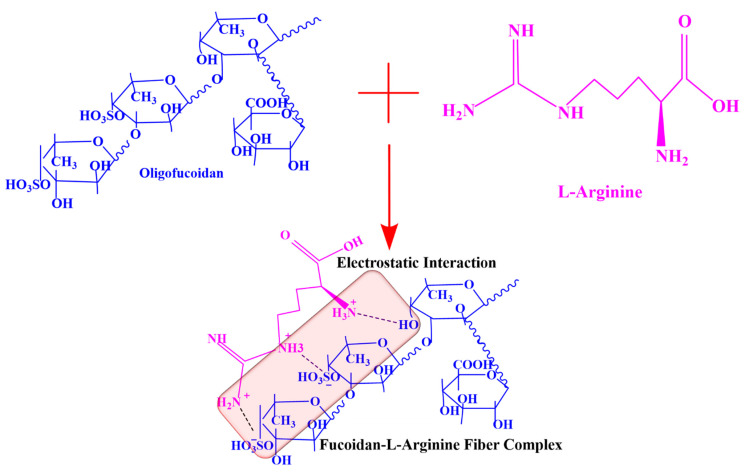
Schematic representation of the electrostatic interaction between fucoidan and L-arginine.

The UV absorbances of fucoidan, L-arginine, and their complexes were carried out to understand the interaction between fucoidan and L-arginine. In the case of oligo-fucoidan, two absorbance peaks were observed at 212 nm and 266 nm. In contrast, in the case of L-arginine, the absorbance peak appeared at a very low wavelength of 205 nm. The absorption peak of the Fuc-L-Arg complex occurred at lower wavelengths as the L-arginine concentration increased, including 220 nm and 266 nm (1:1), 213 nm and 264 nm (1:2.5), and 208 nm and 262 nm (1:5), respectively (Table 1) [24]. Thus, blueshift of the spectrum in the Fuc-L-Arg complex arises due to the H-aggregation of the L-arginine appearing upon the fucoidan surface [35] (Figure 1a). The corresponding FT-IR spectrum of fucoidan was 3271 cm^−1^ and 1604 cm^−1^, which indicates the presence of OH and C=O in uronic acid groups. The peaks around 1417 cm^−1^ and 891 cm^−1^, 1251 cm^−1^, and 1024 cm^−1^ indicate the presence of C–O–S, S=O, and saccharide groups, respectively [4,15,22,24]. The FT-IR spectrum of pristine L-arginine characteristic bands is the broad band of NH and OH stretching vibrations appearing at 3271 cm^−1^ and 3049 cm^−1^. In addition, the peaks around 2943 cm^−1^ and 2861 cm^−1^ appear to be the stretching vibration of CH. The peaks at 1674 cm^−1^, 1612 cm^−1^, and 1551 cm^−1^ represent the bending vibrations of NH_2_ and COO^−^. In addition, the peaks at 1474 cm^−1^ and 1420 cm^−1^ indicate the deformation vibrations of CH and NH and the presence of the symmetrical bending vibration of CH_3_, respectively. Further, the peak around 1331 cm^−1^ indicates the presence of the bending vibration of OH. The peaks around 1183 cm^−1^ and 1131 cm^−1^ represent the stretching vibration of CO and the deformation vibration of OH as well as the stretching vibration of CN, respectively. The peaks around 974 cm^−1^, 765 cm^−1^, and 700 cm^−1^ reflect the presence of the OH stretching vibration, CH deformation vibration, and COO^−^ bending vibration, respectively [21,25,30]. In the Fuc-L-Arg complex, the broad band that appears from 3000–3700 cm^−1^ represents the presence of the stretching vibrations of NH and OH from both fucoidan and L-arginine. In addition, the peaks at 2974 cm^−1^ and 2902 cm^−1^ indicate the presence of the L-arginine CH stretching vibration. The α-helical electrostatic interaction between the fucoidan sulfate group and the L-arginine amine group was confirmed through the shifting of amine peaks and the C–O–S peak to a lower wavenumber from 1612 cm^−1^ to 1600 cm^−1^ and 1588 cm^−1^, and 1417 cm^−1^ to 1414 cm^−1^ and 1406 cm^−1^, respectively. In addition, the saccharide group redshift appears from 1020 cm^−1^ to 1032 cm^−1^, and 1048 cm^−1^ occurs in the 1:1 and 1:2:5 complexes, indicating the electrostatic interaction of the sulfate-bonded saccharide group with the amine group of L-arginine (Figure 1b, Table 1). Additionally, NMR data are included in the supplementary information (Supplementary Figure S1). The electrostatic interaction between fucoidan and L-arginine is represented via a structural schematic diagram (Figure 2).

### 3.2. Morphology of the Fuc-L-Arg Fiber Complex and Its Impact on Cellular Uptake

**Figure 3:**
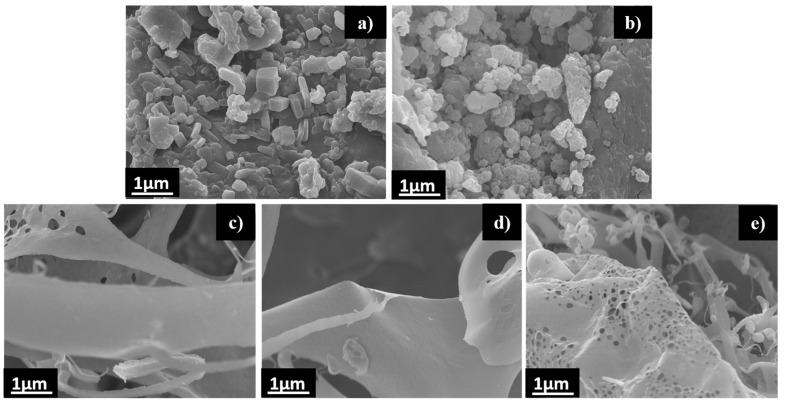
FE-SEM morphology: (**a**) pristine fucoidan, (**b**) pristine L-arginine, (**c**) Fuc-L-Arg (1:1) complex, (**d**) Fuc-L-Arg (1:2.5) complex, and (**e**) Fuc-L-Arg (1:5) complex.

The morphologies of the pristine fucoidan, pristine L-arginine, and Fuc-L-Arg (1:1, 1:2.5, and 1:5) complexes were observed using FE-SEM. Both pristine fucoidan and L-arginine exhibited aggregate structures, whereas, in the case of Fuc-L-Arg, a porous sheet-like fiber complex was observed (Figure 3d). This is likely due to the α-helical electrostatic interaction of the fucoidan sulfate group with the L-arginine amine group [12]. Further, the energy-dispersive X-ray spectroscopy confirmation of elements is presented in the supplementary data (S2). Fiber morphology plays a significant role in cellular internalization through vertical overlap with the cell membrane [36,37] (Figure 3).

### 3.3. Confirmation of Fucoidan Conjugation to Cy3 Dye Using Fluorescent Spectroscopy Techniques

**Figure 4:**
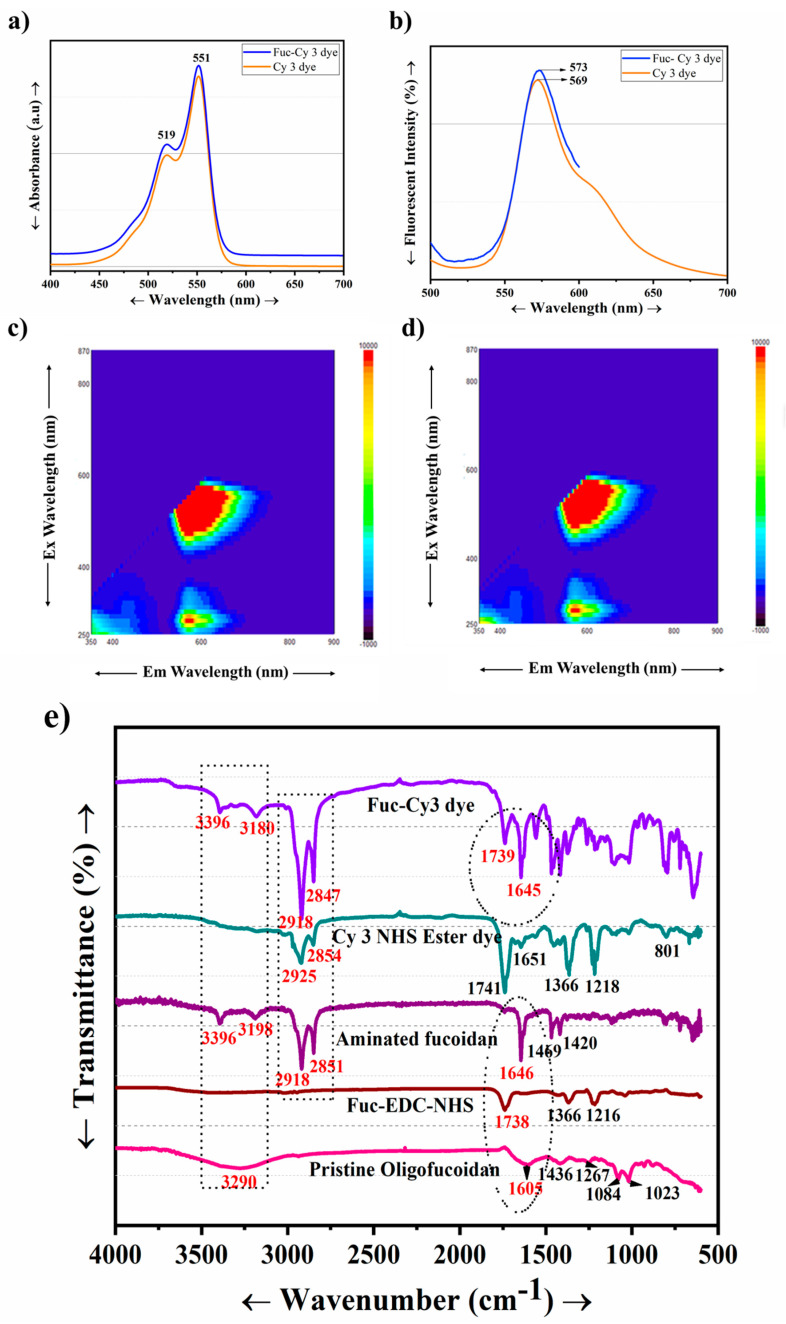
(**a**) UV absorbances of the Cy3 NHS ester and Fuc-Cy3 dyes, (**b**) two-dimensional fluorescence intensities of the Cy3 NHS ester and Fuc-Cy3 dyes, (**c**) three-dimensional fluorescence intensity of the Cy3 NHS ester dye, (**d**) three-dimensional fluorescence intensity of the Fuc-Cy3 dye, (**e**) FT-IR studies of the Fuc-Cy3 fiber complex.

**Figure 5:**
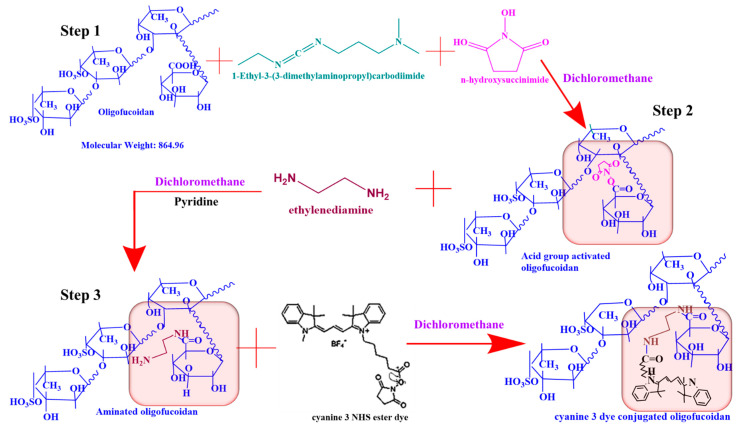
Schematic representation of the conjugation of Cy3 dye with Fucoidan (Fuc-Cy 3).

Fucoidan is a non-fluorescent hetero-polysaccharide. Hence, we conjugated Cy3 dye to fucoidan to track cellular internalization of the Fuc-L-Arg fiber complex via red fluorescence. Conjugation of the Fuc-Cy3 complex was confirmed using UV fluorescence intensity, FT-IR, and NMR spectroscopic techniques. Accordingly, we confirmed the conjugation of Fuc-Cy3 using UV spectroscopy at a maximum absorbance level of 551 nm. Further, Fuc-Cy3 exhibited a slight blueshift in fluorescence to 569 nm (lower wavenumber) from 573 nm (higher wavenumber) of the Cy3 dye (Figure 4a,b). Additionally, the three-dimensional fluorescence intensity revealed an excellent conjugation of Fuc-Cy3 dye with acceptable fluorescence intensity of the Cy3 dye (Figure 4c,d). Furthermore, the conjugation of fucoidan to the Cy3 dye was confirmed using FT-IR spectroscopy. The peaks at 1605 and 3280 cm^−1^ indicate the presence of C=O and OH groups in oligo-fucoidan, respectively, which occur when these acid groups are activated using EDC and NHS. The ester peak around 1738 cm^−1^ represents the formation of an activated acid group by the formation of ester bonds with NHS. Once EDA and pyridine were added, the activated acid group was confirmed by the peaks around 3386 and 3187 cm^−1^ for symmetric and asymmetric stretching amine groups, respectively, in addition to the increased transmittance of the carbonyl group at 1648 cm^−1^. In pristine Cy3 NHS ester, the strongest peaks were found to occur around 1731 cm^−1^. The strong carbonyl group peak at 1638 cm^−1^ clearly indicated the presence of a carbonyl group. The peaks at 3392 cm^−1^ and 3177 cm^−1^ indicate the presence of amine groups (Figure 4e). Therefore, successful conjugation of aminated fucoidan to Cy3 dye was confirmed. In addition, NMR data of Fuc-Cy3 dye conjugation are presented in the supplementary data (S3). A structural schematic representation of the Fuc-Cy3 interaction is shown in Figure 5.

### 3.4. UV Spectral Confirmation of the Fuc-Cy3-L-Arg Fiber Complex

**Figure 6:**
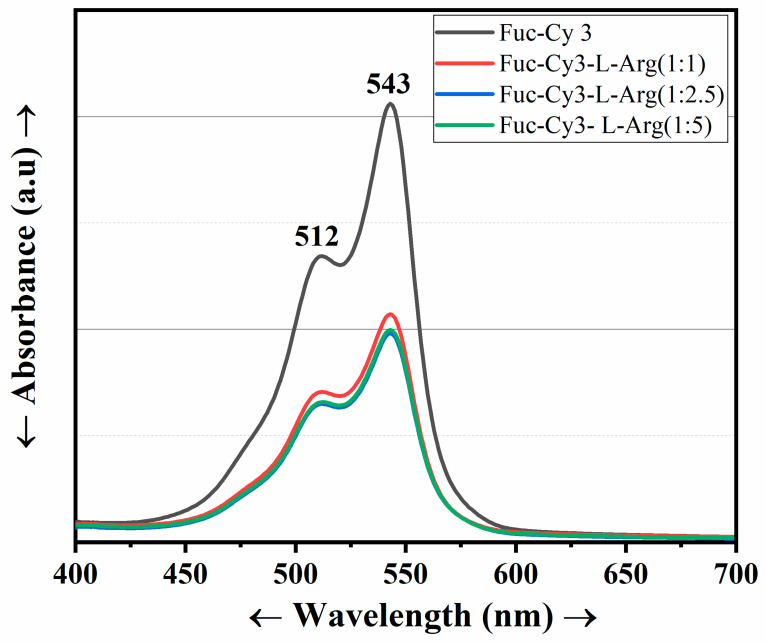
Fluorescence intensities of the Fuc-Cy3 dye and Fuc-Cy3-L-Arg (1:1, 1:2.5, and 1:5) complexes.

Prior to cellular internalization, the Fuc-Cy3-L-Arg fiber complex absorbances and intensities were observed using UV and photoluminescent spectroscopy, respectively. The Cy3 dye was then conjugated to fucoidan to assess cellular uptake. The UV absorbances of the Fuc-Cy3-L-Arg complexes (1:1, 1:2.5, and 1:5) were measured and determined to be similar to that of Fuc-Cy3 at 512 and 543 nm(Figure 6) [24,38]. The fluorescence intensities of the Fuc-Cy3-L-Arg complexes are shown in the supplementary data (S4). Thereafter, the confirmed Fuc-Cy3-L-arg complexes were used for further biocompatibility and cellular internalization studies.

### 3.5. Biocompatibility Test of the Fuc-L-Arg Fiber Complex in HeLa Cells

**Figure 7:**
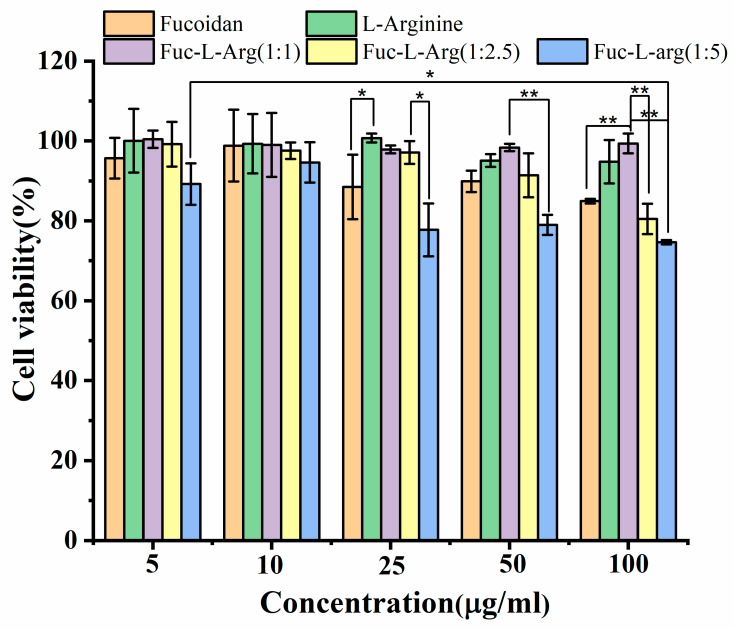
Cell viabilities of pristine fucoidan, pristine L-arginine, and the Fuc-L-Arg complexes (1:1, 1:2.5, and 1:5) in HeLa cells. Where * represents *p* < 0.05, ** *p* < 0.01.

Prior to in vitro and in vivo studies of the material, the cytotoxicity of the biomaterial was a significant factor for consideration. Generally, fucoidan exhibits non-toxicity due to its varied biological properties and wide bioapplications in the food and pharmaceutical industries [22,39]. Previous reports have found that L-arginine can exhibit >80% biocompatibility up to 35 mg [34]. In this study, biocompatibility tests were carried out for pristine oligo-fucoidan, pristine L-arginine, and the Fuc-L-Arg complex prior to cellular uptake. The cell viabilities were >80% for various concentrations (5, 10, 25, 50, 100 µg/mL obtained from 1 mg/mL stock concentration) in pristine oligo-fucoidan and L-arginine as well as Fuc-L-Arg fiber (1:1, 1:2.5) complexes. In addition, Fuc-L-Arg (1:5) complexes also exhibited >80% cell viability at lower concentrations (5 and 10 µg/mL), whereas at higher concentrations (25, 50, 100 µg/mL), the Fuc-L-Arg (1:5) complex exhibited <80% cell viability (Figure 7). This is due to higher internalization of the complex into the HeLa cell, and leads to the exhibition of its potential biological activities like anti-cancer activity. Further, the statistical analysis was carried out at higher concentrations such as 25, 50, and 100 µg/mL between fucoidan, L-arginine, and Fuc-L-Arg complexes (1:1, 1:2.5, and 1:5). There is a significant difference of about *p* < 0.05 between fucoidan and L-arginine and Fuc-L-Arg complexes (1:2.5 and 1:5) at the concentration of 25 µg/mL; whereas at the concentration of 50 µg/mL, between 1:1 and 1:5 Fuc-L-Arg complexes, the significant difference is about *p* < 0.01. In the case of 100 µg/mL, there is significant difference of about *p* < 0.01 between Fuc-L-Arg (1:1) and fucoidan, Fuc-L-Arg (1:1) and Fuc-L-Arg (1:2.5), and Fuc-L-Arg (1:1) and Fuc-L-Arg (1:5). From the above findings, we conclude that as the Fuc-L-Arg complex concentration increases, Fuc-L-Arg (1:5) shows cell viability with a significant difference of *p* < 0.05 and 0.01 compared to the other two complexes (1:1 and 1:2.5). This is because of higher internalization of the Fuc-L-Arg complex (1:5) as the L-arginine weight ratios increased and started exhibiting fucoidan biological activities like anti-cancer activity.

### 3.6. Qualitative Analysis of the Cellular Uptake of Fuc-Cy3 and the Fuc-Cy3-L-Arg Fiber Complex in HeLa Cells

**Figure 8:**
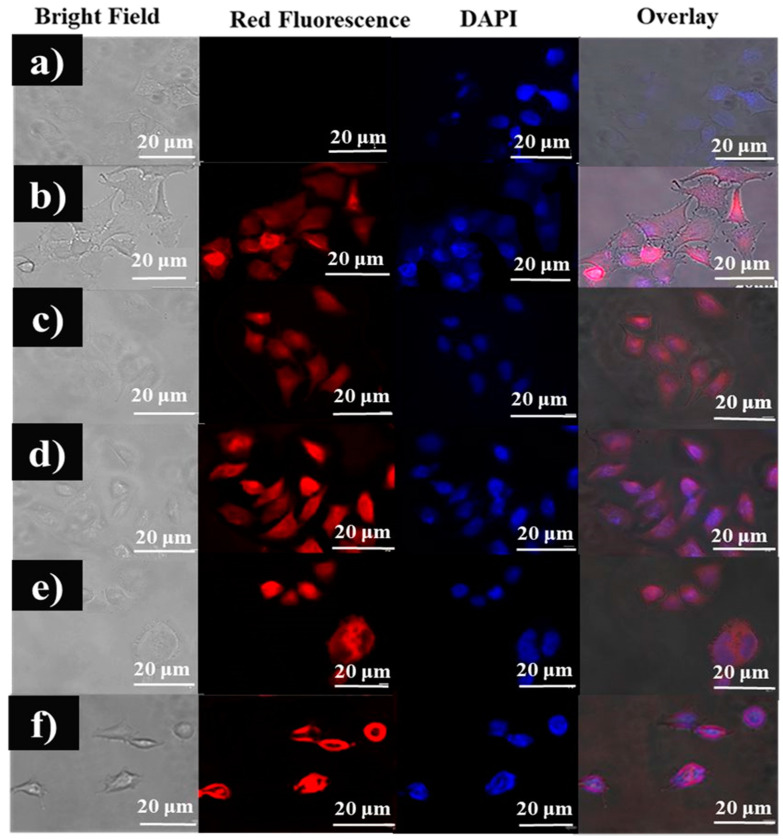
Cellular uptake of (**a**) control HeLa cells, (**b**) Cy3-treated HeLa cells, (**c**) Fuc-Cy3-treated HeLa cells, (**d**) Fuc-Cy3-L-Arg (1:1)-treated HeLa cells, (**e**) Fuc-Cy3-L-Arg (1:2.5)-treated HeLa cells, and (**f**) Fuc-Cy3-L-Arg (1:5)-treated HeLa cells.

Moreover, the anionic polysaccharides are effective in binding to the cellular receptors at certain levels [40], which could be organized as a suitable formulation to increase permeability at a specific absorption site. To investigate cellular internalization of the Fuc-L-Arg complex, the complex was incubated with HeLa cells for 4 h and observed through red fluorescence obtained from Cy3 dye and blue fluorescence obtained due to nuclei staining obtained by DAPI dye using fluorescence microscopy (Figure 8). Fucoidan, a negatively charged polysaccharide, has less intense cellular internalization; however, cellular internalization is enhanced when formulated with L-arginine (a positively charged polysaccharide). The zeta potential charge of the Fuc-L-Arg complex was studied at physiological pH (7.4). The surface charge of the Fuc-L-Arg complex at physiological pH changes to positive as the L-arginine weight ratio increases. This improved cellular internalization may be due to a reduction in the negative surface charge of fucoidan when formulated with L-arginine (Table 2). Therefore, the Fuc-L-Arg fiber complex displayed greater cellular uptake in HeLa cells.

### 3.7. Quantitative Flow Cytometry of the Fuc-Cy3 Dye and Fuc-L-Arg Fiber Complex in HeLa Cells

**Figure 9:**
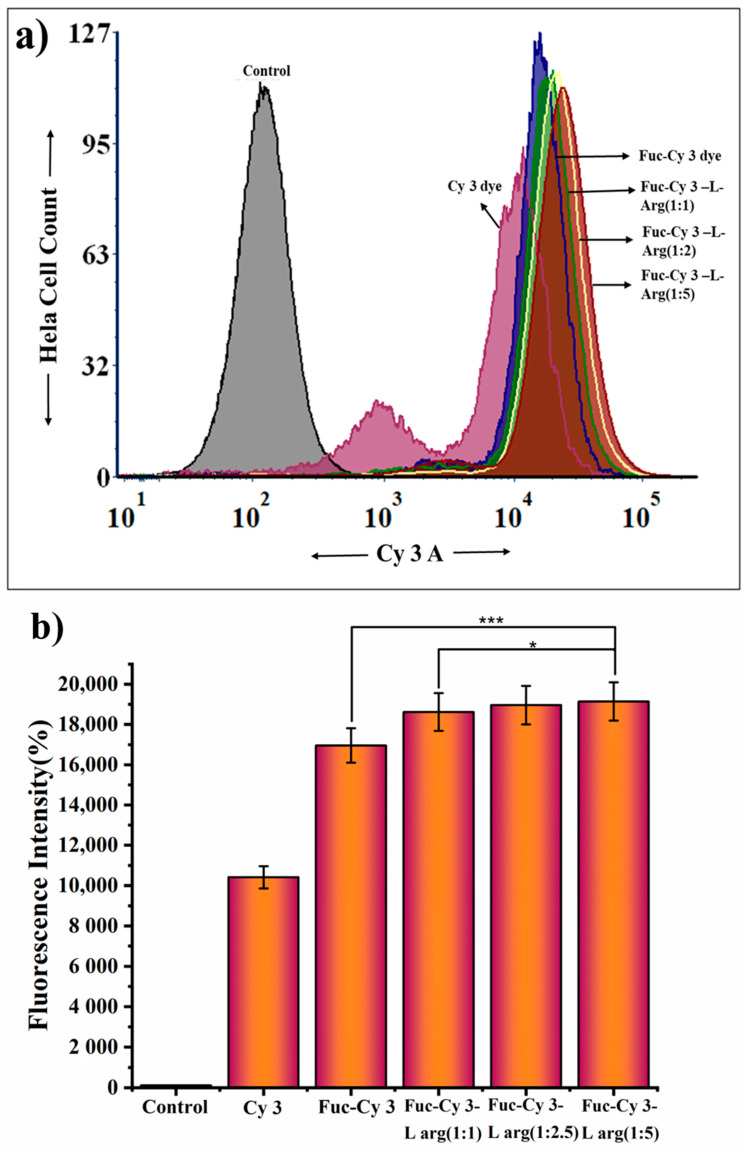
Quantitative flow cytometry analysis of (**a**,**b**) fluorescence intensity of control HeLa cells (without treatment), Cy3 dye-treated HeLa cells, Fuc-Cy3 dye-treated HeLa cells, Fuc-L-Arg (1:1)-treated HeLa cells, Fuc-L-Arg (1:2.5)-treated HeLa cells, and Fuc-L-Arg (1:5)-treated HeLa cells. Where * represents *p* < 0.05 and *** represents *p* < 0.001.

Quantitative flow cytometry was used to study the cellular internalization of the Fuc-L-Arg complex in HeLa cells. Untreated HeLa cells were used as negative controls. In contrast, Cy3 dye-treated cells were considered as positive controls. Based on the positive control fluorescence intensity, the quantitative cellular internalization of the Fuc-Cy3 dye and Fuc-Cy3-L-Arg complex was compared. Compared to pristine oligo-fucoidan, the Fuc-L-Arg fiber complex showed increased cellular internalization. As the L-arginine concentration increased, the quantitative cellular internalization of oligo-fucoidan also increased (Figure 9a,b). The above findings were confirmed by increased fluorescence intensity of the Cy3 dye in each complex. The statistical analysis was carried out for Figure 9b between Fuc-Cy3 dye, Fuc-L-Arg (1:1) and Fuc-L-Arg (1:5). From the analysis, we found very strong statistical difference between Fuc-Cy3 dye-treated and the Fuc-L-Arg (1:5) complex-treated HeLa cells of about *p* < 0.001. Furthermore, we found statistical difference between the Fuc-L-Arg (1:1) and Fuc-L-Arg (1:5) complexes of about *p* < 0.05. In contrast, the difference between the Fuc-L-Arg (1:2.5) and Fuc-L-Arg (1:5) complexes was non-significant. This is because the fluorescence intensity of the two Fuc-L-Arg complexes (1:2.5 and 1:5) were within the limits of fluorescence intensity values (Table 3). From the fluorescence intensity values in Table 3, we also found that compared to all three complexes, the Fuc-L-Arg (1:5) complex exhibited slightly higher intensity values. Hence, we highlight that the Fuc-L-Arg (1:5) fiber complex has improved cellular internalization than the other two Fuc-L-Arg complexes as the weight of the complex increases.

## 4. Conclusions

The presence of sulfate groups in fucoidan results in a negative surface charge and presents a barrier to cellular internalization. In our study, an electrostatically interacting Fuc-L-Arg fiber complex was prepared and an enhanced cellular internalization was observed for the Fuc-L-Arg complex compared to pristine oligo-fucoidan. We have also found that as the weight ratio of Fuc-L-Arg increased, cellular internalization was improved. The above outcome is due to the electrostatic interaction of L-arginine between oligo-fucoidan and the cell membrane. Hence, this finding provides additional evidence that enhanced oligo-fucoidan internalization can improve the potential biological activities of fucoidan, such as anti-cancer and anti-oxidant activities.

## Figures and Tables

**Table 1:** UV absorbance and FT-IR characteristic peaks in Fucoidan, L-Arginine, and Fuc-L-Arg fiber complex.

Compound	UV Absorbance Peaks	FT-IR Characteristic Peaks
Fucoidan	212 nm and 266 nm	3315 (OH)
		1614 (C=O)
		1429 and 891 (C−O−S)
		1160–1259 (S=O) and 1021 (Saccharide group)
L-arginine	205 nm	3282 and 3106 (NH and OH)
		2928 and 2862 (CH)
		1742 (COO−)
Fuc-L-Arg fiber complex	220 nm and 266 nm (1:1)	3325 (OH)
	213 nm and 264 nm (1:2.5)	2928 and 2862 (CH)
	208 nm and 262 nm (1:5)	1742 (COO−)

**Table 2:** Zeta potential charge measurement without and with the Cy3 dye complex.

S. No.	Oligo-Fucoidan	L-Arginine	Fuc-L-Arg (1:1)	Fuc-L-Arg (1:2.5)	Fuc-L-Arg (1:5)
Without Cy3 dye (pH 7.4)	−139.6	1.7	−4.4	−2.7	6.1
With Cy3 dye (pH 7.4)	−76.6	0.7	−7.1	−1.6	5.5

**Table 3:** Fluorescence intensity of different complexes in HeLa cells.

Groups	Control	Cy3 Dye	Fuc-Cy3	Fuc-L-Arg (1:1)	Fuc-L-Arg (1:2.5)	Fuc-L-Arg (1:5)
Cy3 Dye Intensity	109	10,412	16,955.33333	18,617.66667	18,961	19,142

## Supplementary Materials

The following are available online at https://www.mdpi.com/article/10.3390/polym13111795/s1, Figure S1. Nuclear Magnetic Resonance of pristine fucoidan, pristine L-Arginine and varied ratios of Fuc -L-Arg Complex (1:1,1:2.5 and 1:5). Figure S2. EDAX measurement of pristine Oligo-fucoidan, pristine L-Arginine, Fucoidan-L-arginine (1:1, 1:2.5 & 1:5). Figure S3. Nuclear Magnetic Resonance of fucoidan-Cy 3 dye. Figure S4. Fluorescent Intensity and FT-IR Spec-trum of varied ratios of Fuc-Cy 3-L-Arg (1:1,1:2.5 &1:5) complex.
