# Supplementary material for: Enhanced Cellular Uptake in an Electrostatically Interacting Fucoidan–L-Arginine Fiber Complex
Source: Polymers (Basel). 2021 May 29;13(11):1795. doi: 10.3390/polym13111795 (PMC8198147; doi:10.3390/polym13111795)
Supplement: Supplementary file 1 [file polymers-13-01795-s001.zip › polymers-1184288-supplementary.pdf]

polymers

Supplementary Materials: Enhanced Cellular Uptake in an Electrostatically Interacting Fucoidan-L-Arginine Fiber Complex

Vinothini Arunagiri [1], Hsieh-Chih Tsai [1,2,3]*, Haile Fentahun Darge [1,4], Endiries Yibru Hanurry [1], Chang Yi Lee [1], Juin-Yih Lai [1,2,3] and Szu-Yuan Wu [5,6,7,8,9,10,11]*

1 Graduate Institute of Applied Science and Technology, National Taiwan University of Science and Technology, Taipei 106, Taiwan;
2 Advance Membrane Materials Center, National Taiwan University of Science and Technology, Taipei 106, Taiwan
3 R&D Center for Membrane Technology, Chung Yuan Christian University, Chungli, Taoyuan 320, Taiwan
4 College of Medicine and Health Science, Bahir Dar University, Bahir Dar 79, Ethiopia
5 Department of Food Nutrition and Health Biotechnology, College of Medical and Health Science, Asia University, Taichung 413, Taiwan
6 Big Data Center, Lo-Hsu Medical Foundation, Lotung Poh-Ai Hospital, Yilan 256, Taiwan
7 Division of Radiation Oncology, Department of Medicine, Lo-Hsu Medical Foundation, Lotung Poh-Ai Hospital, Yilan 256, Taiwan
8 Department of Healthcare Administration, College of Medical and Health Science, Asia University, Taichung 413, Taiwan
9 Cancer Center, Lo-Hsu Medical Foundation, Lotung Poh-Ai Hospital, Yilan 256, Taiwan
10 Graduate Institute of Business Administration, Fu Jen Catholic University, Taipei 242, Taiwan
11 Centers for Regional Anesthesia and Pain Medicine, Taipei Municipal Wan Fang Hospital, Taipei Medical University, Taipei 110, Taiwan
* Correspondence: h.c.tsai@mail.ntust.edu.tw (H.C.T.); szuyuanwu5399@gmail.com (S.-Y.W.);
Tel.: +886-2-27303779 (H.C.T.)

1. NMR result of fuc-l-arginine complex

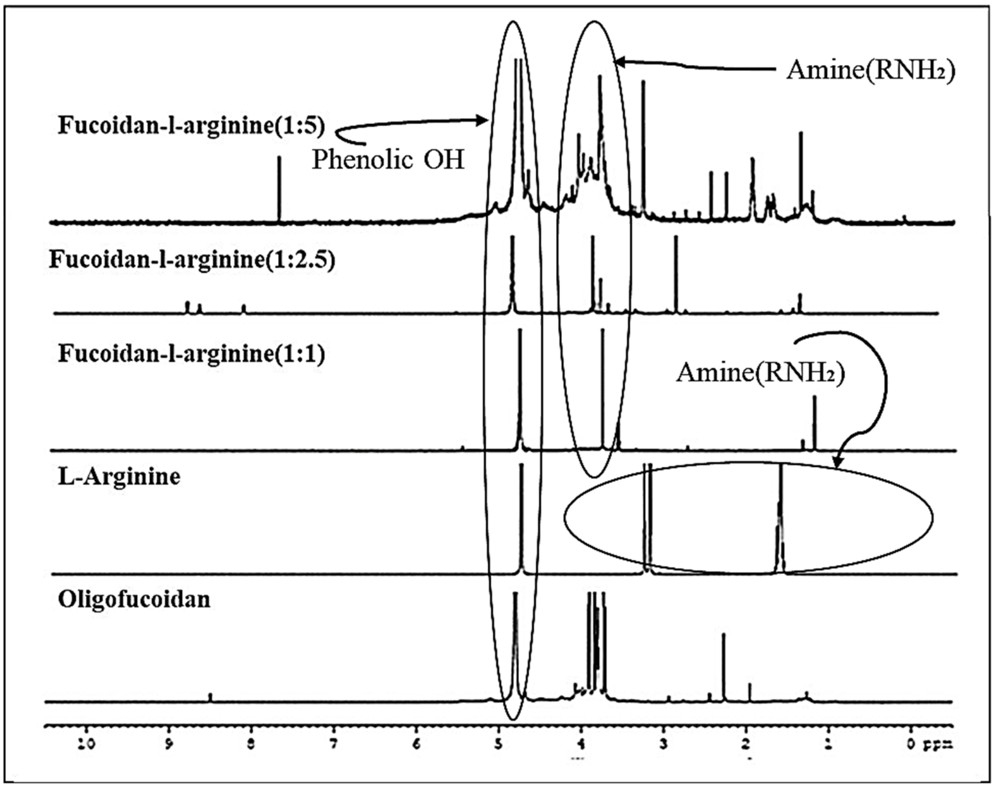

Figure S1. Nuclear Magnetic Resonance of pristine fucoidan, pristine L-Arginine and varied ratios of Fuc -L-Arg Complex (1:1,1:2.5 and 1:5).

H^1 NMR was carried out for pristine Oligofucoidan, L-Arginine & Fuc-L-Arg (1:1,1:2.5&1:5) complex in D_2O solvent. Oligofucoidan exhibits aromatic-CH proton H^1 NMR peak at 2.3ppm, -O-CH_2 proton H^1 NMR at 3.9 to 4 ppm and phenolic OH H^1 NMR peak at 4.9 ppm. Further, L-Arginine comprises amine group proton H^1 NMR at 1.5 &3.3 ppm and phenolic OH H^1 NMR peak at 4.9 ppm. Whereas, the Fuc-L-Arg(1:1) exhibits shift of one amine group proton H^1 NMR peak at 3.9ppm indicates the interaction of amine group proton H^1 of l-arginine with sulphate group of oligofucoidan. Further, the phenolic OH H^1NMR peak at 4.9 ppm were unaltered in the complex (1:1). Further, as the l-arginine concentration increases, the interaction between sulfate group(oligofucoidan) and amine group proton H^1 NMR increases. Hence, we can able to find broader and slightly improved shift of amine group proton H^1 NMR to 4ppm with unaltered shift of phenolic OH H^1NMR peak from 4.9 ppm.

2. EDAX Measurement:

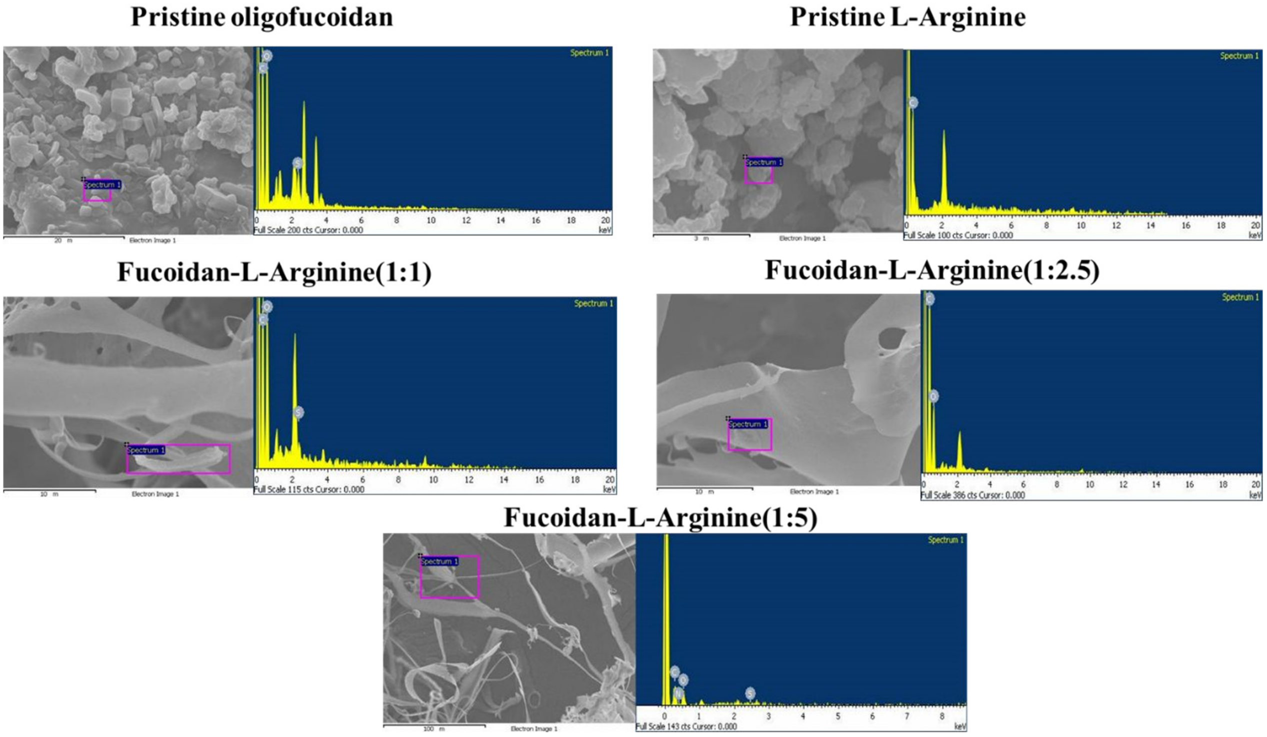

Figure S2. EDAX measurement of pristine oligo-fucoidan, pristine L-Arginine, Fucoidan-L-arginine (1:1, 1:2.5 & 1:5).

EDAX measurement of pristine fucoidan, l-arginine and fucoidan –l-arginine was carried out to study the interaction between fucoidan and L-Arginine. In fucoidan, the element presented were C, O and S group. In case of L-Arginine, the element presented were C, O and N. Further, the presence of C, N, O and S element in Fuc-L-Arg (1:1,1:2.5 &1:5) confirms the interaction between fucoidan and L-Arginine.

3. NMR result of Fuc-Cy 3 Conjugated dye:

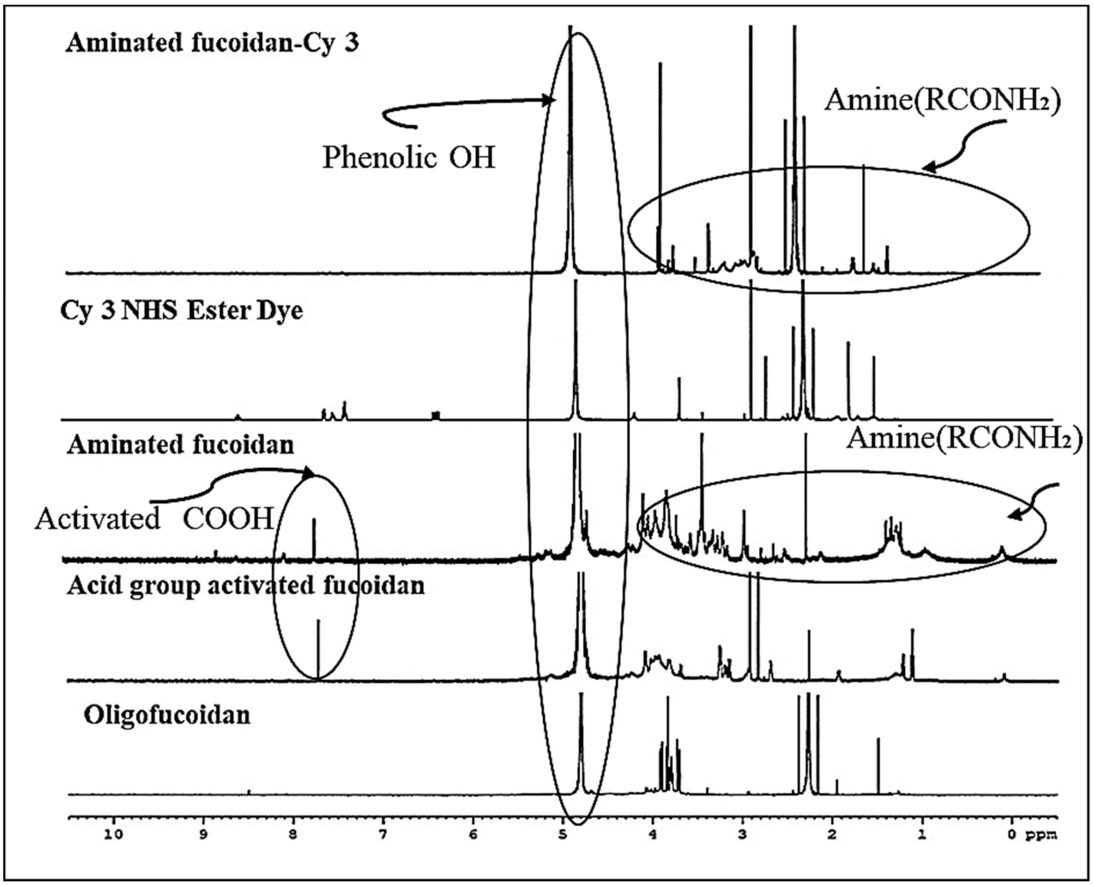

Figure S3. Nuclear Magnetic Resonance of fucoidan-Cy 3 dye.

H^1 NMR was carried out for oligofucoidan, cy 3 dye and Fuc-cy 3 dye in order to confirm the interaction between fucoidan and Cy 3 dye. Oligo-fucoidan exhibits aromatic-CH proton H^1 NMR peak at 2.3ppm, -O-CH_2 proton H^1 NMR at 3.9 to 4 ppm and phenolic OH H^1 NMR peak at 4.9 ppm. Further, Cy 3 dye exhibits phenolic OH H^1 NMR peak at 4.9 ppm, aromatic-H-C=O proton H^1 NMR peak at 2-3ppm and Aromatic-HC=CH-Aromatic proton H^1 NMR peak at 1-2ppm.Further, activated acid group oligofucoidan by adding an EDC NHS was proved by an extra peak near to 8ppm which indicates H^1NMR proton of activated acid group in oligofucoidan. Further, when EDA added to the activated acid group, broader peak was appeared near 2-4ppm indicated the presence of amine group and carbonyl group ie.amination. Additionally, there was a shorter peak at 8ppm indicates some unreacted carboxylic group. Furthermore, we observed the strong peak from 2 to 4ppm clearly indicates the presence of amine group and carbonyl group ie. aromatic amide bond presence in the final aminated fucoidan-Cy 3 dye complexes with disappearance of acid group peak at 8ppm.

4. Fluorescence intensity and FT-IR spectrum of Fuc-cy 3-L-Arg Complex:

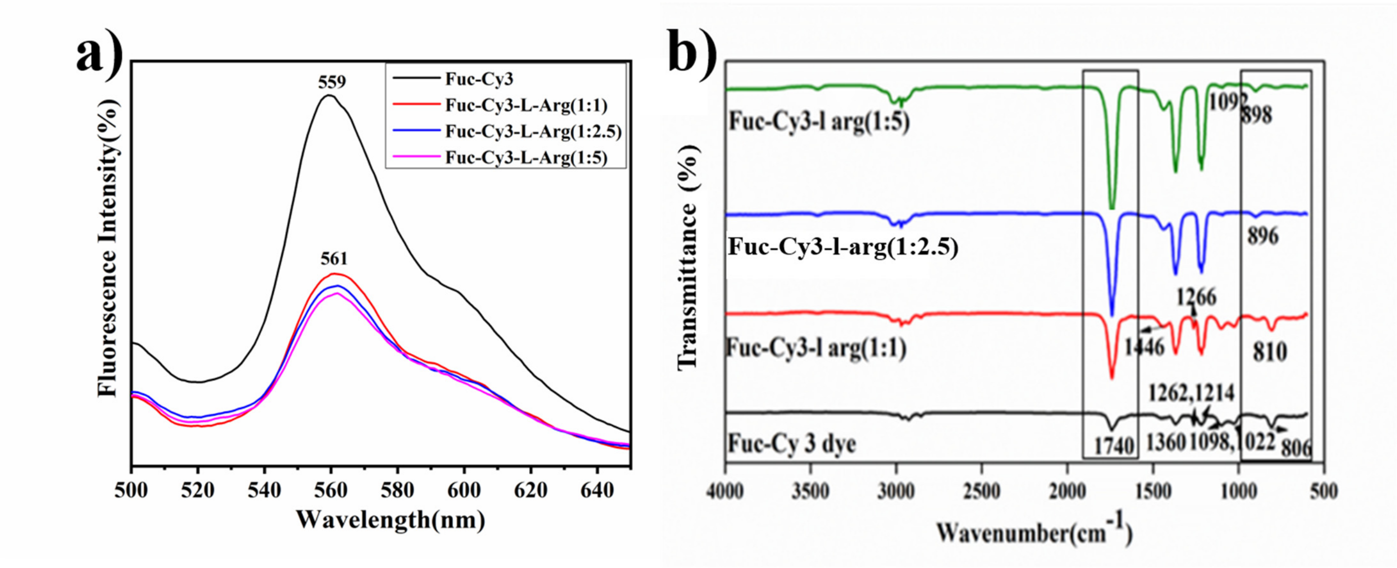

Figure S4. Fluorescent Intensity and FT-IR Spectrum of varied ratios of Fuc-Cy 3-L-Arg (1:1,1:2.5 &1:5) complex.

The FT-IR of Fuc-Cy3 dye and Fuc-L-Arg(1:1), (1:2.5) & (1:5) complex were studied to represent that conjugation of cy 3 dye to fucoidan have left unaltered the binding site of l-arginine with fucoidan. Further, Fuc-L-Arg complex have exhibited the slight difference in fluorescent property after cy 3 dye conjugation. This occurs owing to the quenching property of dye occurs as the l-arginine conc. increases.
